# Suberin, the hallmark constituent of bark, identified in a 45-million-year-old monkeyhair tree (*Coumoxylon hartigii*) from Geiseltal, Germany

**DOI:** 10.1038/s41598-023-50402-y

**Published:** 2024-01-02

**Authors:** Mariam Tahoun, Carole T. Gee, Victoria E. McCoy, Michael Stoneman, Valerica Raicu, Marianne Engeser, Christa E. Müller

**Affiliations:** 1 Department of Pharmaceutical and Medicinal Chemistry, PharmaCenter Bonn, Pharmaceutical Institute, University of Bonn, An der Immenburg 4, 53121 Bonn, Germany; 2 Division of Paleontology, Institute of Geosciences, University of Bonn, Nussallee 8, 53115 Bonn, Germany; 3 Department of Geosciences, University of Wisconsin-Milwaukee, Milwaukee, WI 53211 USA; 4 Department of Physics, University of Wisconsin-Milwaukee, Milwaukee, WI 53211 USA; 5 Department of Biological Sciences, University of Wisconsin-Milwaukee, Milwaukee, WI 53211 USA; 6 Kekulé Institute for Organic Chemistry and Biochemistry, University of Bonn, 53121 Bonn, Germany

**Keywords:** Plant sciences, Biogeochemistry, Chemistry

## Abstract

Suberin, a complex biopolymer, forms a water- and gas-insoluble barrier that protects the inner tissues of plants. It is abundant in tree bark, particularly in the cork oak *Quercus suber*. Anatomically, fossil bark has been described since the Devonian. However, its distinctive constituent suberin has not yet been reported from the fossil record. Here we present unambiguous chemical evidence for intact suberin from the bark of a middle Eocene monkeyhair tree from Geiseltal, eastern Germany. High-performance liquid chromatography coupled to electrospray ionization mass spectrometry (HPLC–ESI-MS) detected constituents of suberin in the outer layer the fossil monkeyhair tree, which confirms previous morphological interpretation of this tissue as bark, and chemically differentiates this layer from the two tissues of the inner layer. Notably, this is the first study with compelling chemical evidence for suberin in fossil bark. Fluorescence microspectroscopy additionally supports the presence of suberin. Fossilization conditions in the Eocene Geiseltal deposit were likely mild, with low moisture and temperatures, contributing to the remarkable preservation of bark and inner laticifer mats of the monkeyhair trees growing there 45 million years ago.

## Introduction

Suberin is a complex biopolymer that is essential to all woody plants. It is water and gas insoluble and provides a physical and chemical barrier that protects the inner tissues of the tree from loss of water, infection by microbes, and heat exposure. While also found in the endodermis of roots, the bundle sheath of C_4_ plants, and in some wound tissues, suberin is universal in tree bark. Bark is a non-technical, collective term for the tissues of a woody stem peripheral to the vascular cambium. It is a complex tissue that consists of phloem, cortex, phelloderm, cork cambium (phellogen), and cork (phellem). Suberin occurs in the phellem, the outermost layer of a woody axis, and an abundance of suberin will confirm that this layer is bark.

An economically important source of cork comes from the outer bark of *Quercus suber*, the cork oak tree, which contains 40–50% of suberin in its extraordinarily thick layer of phellem^1^. Major constituents of suberin comprise esters of mainly long-chain (C_16_–C_24_) α,ω-dicarboxylic acids (e.g., 1,18-octadec-9-enedioic acid, 1,20-eicosanedioic acid), and ω-hydroxyacids (e.g., 22-hydroxydocosanoic acid) with glycerol or ferulic acid. Minor components of suberin include 1-alkanols (e.g., 1-tetracosanol) and 1-alkanoic acids (e.g., docosanoic acid)^1,2^.

The majority of angiosperm trees, unlike cork oak, have a thin layer of bark. The thickness of the bark in living trees is usually 1–12 mm, of which 1–2.5 mm is the outer bark^3^. In dead trees, exposed to water, the bark decomposes faster than the underlying wood^4^. This may explain why the occurrence of fossil bark is rare compared to the huge abundance of fossil wood and logs in the geological record^5^. Fossil bark has been reported from the Devonian to the present in progymnosperms, cordaites, conifers, and angiosperms. Fossil bark is generally identified by its cellular structure, when found anatomically preserved^5^.

Highly unusual woody axes have recently been described from the middle Eocene of Geiseltal in eastern Germany, in which bark, but not wood, is preserved^6^. These woody axes are *Coumoxylon hartigii*^7^ and contain mats of well-preserved laticifers which are referred to as monkeyhair or *Affenhaar(e)*^6–9^. Two flattened monkeyhair tree axes were studied. Although they are greatly compressed, they had relatively large diameters indicating that they were either large branches or small to medium-sized, single tree trunks^6^. The preserved monkeyhair tree axes consist of two major tissue layers^6^. The previous identification of bark on the surface of the monkeyhair tree is based on its general resemblance to the bark of living dicot trees and its relative position covering the surface of the trunk. The inner layer consists of naturally vulcanized latex strands arising from laticifers that are embedded in degraded organic matter, which is thought to have been derived from wood. Inside these two layers is a hollow space that was originally likely to have been a central cylinder of solid wood. This poor preservation of wood is also unusual as Eocene wood has been found to preserve hemicellulose and pectin, which are unique and labile biopolymers found in the geological record^10^.

Using morphological analysis, high-resolution X-ray microcomputed tomography (micro-CT), and pyrolysis gas chromatography/mass spectrometry (GC/MS), three distinct tissue types were recognized in the two major layers of the monkeyhair tree axes from the middle Eocene Geiseltal deposit near Halle, eastern Germany^6^. Previous studies have focused on only one tissue in the inner layer, namely, on the morphology, organic chemistry, and geochemistry of the latex strands of laticifers^6,8,11–15^. The outer layer of the Eocene monkeyhair tree axes remains have not been previously studied chemically.

In the present study, we chemically investigated the outer tissue layer as well as two additional tissues from the inner layer of the monkeyhair tree in search for suberin, a distinctive constituent of bark. High-performance liquid chromatography coupled to electrospray ionization mass spectrometry (HPLC–ESI–MS) was used to identify the presence of suberin in the tissue samples. Alkaline hydrolysis of suberin results in the formation of the long-chain fatty acids 1,18-octadec-9-enedioic acid and 1,20-eicosanedioic acids. The occurrence of these abundant fatty acids after depolymerization is used to confirm that the suberin was originally present, in its intact polymeric form, in these samples. In addition, a fluorescence-based approach was used to complement the chemical analysis and provide additional evidence for the presence of suberin.

## Results

### Tissue and extracts

**Figure 1:**
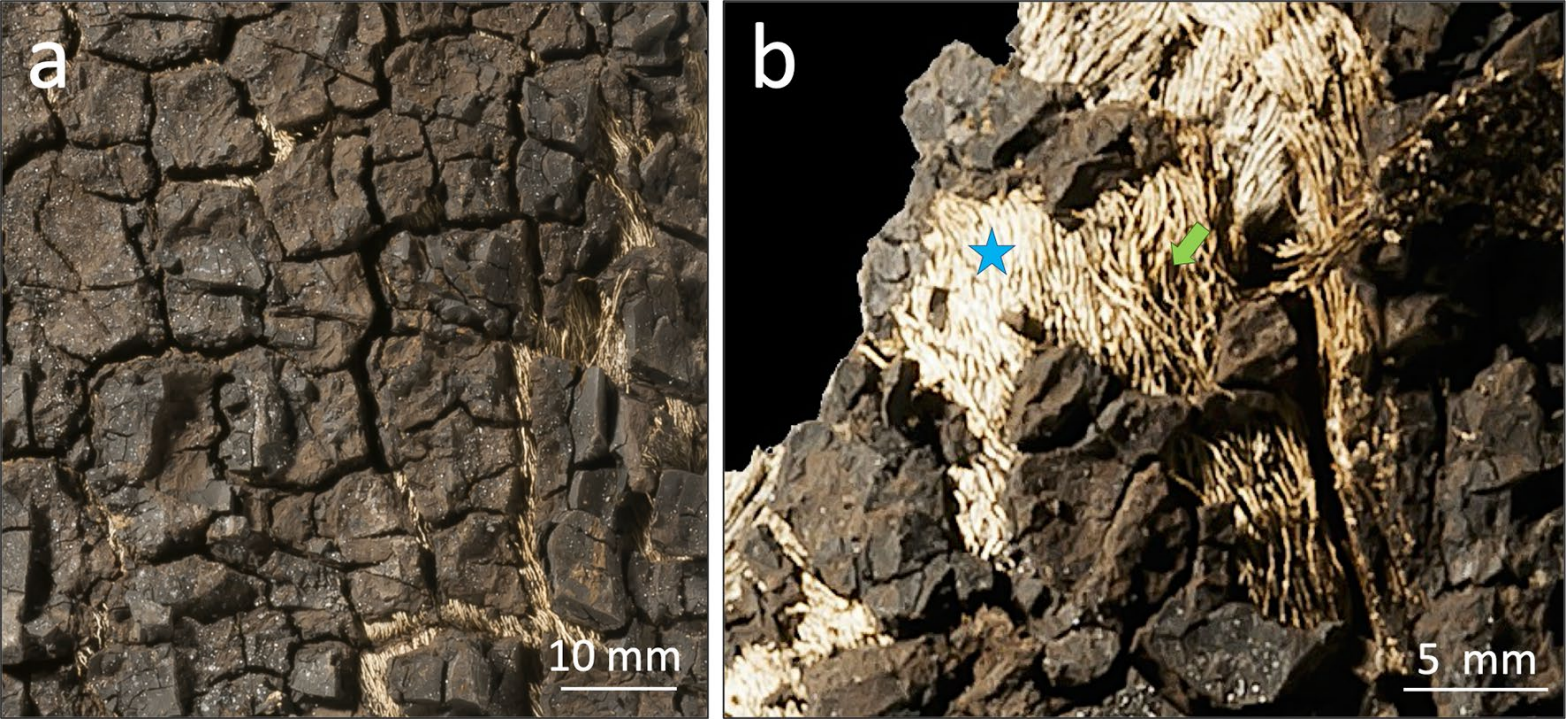
Examples of tissues sampled from the Eocene monkeyhair tree, inventory number GMH Y74. (**a**) Outer layer interpreted as bark (sample I), showing the underlying layer with light-colored laticifer strands. (**b**) Inner layer showing the dark-colored, pasty organic matter thought to derive from degraded wood that embeds the laticifers (green arrow; sample II), as well as the light-colored strands of laticifers themselves (blue star; sample III). Photos taken by Georg Oleschinski.

**Figure 2:**
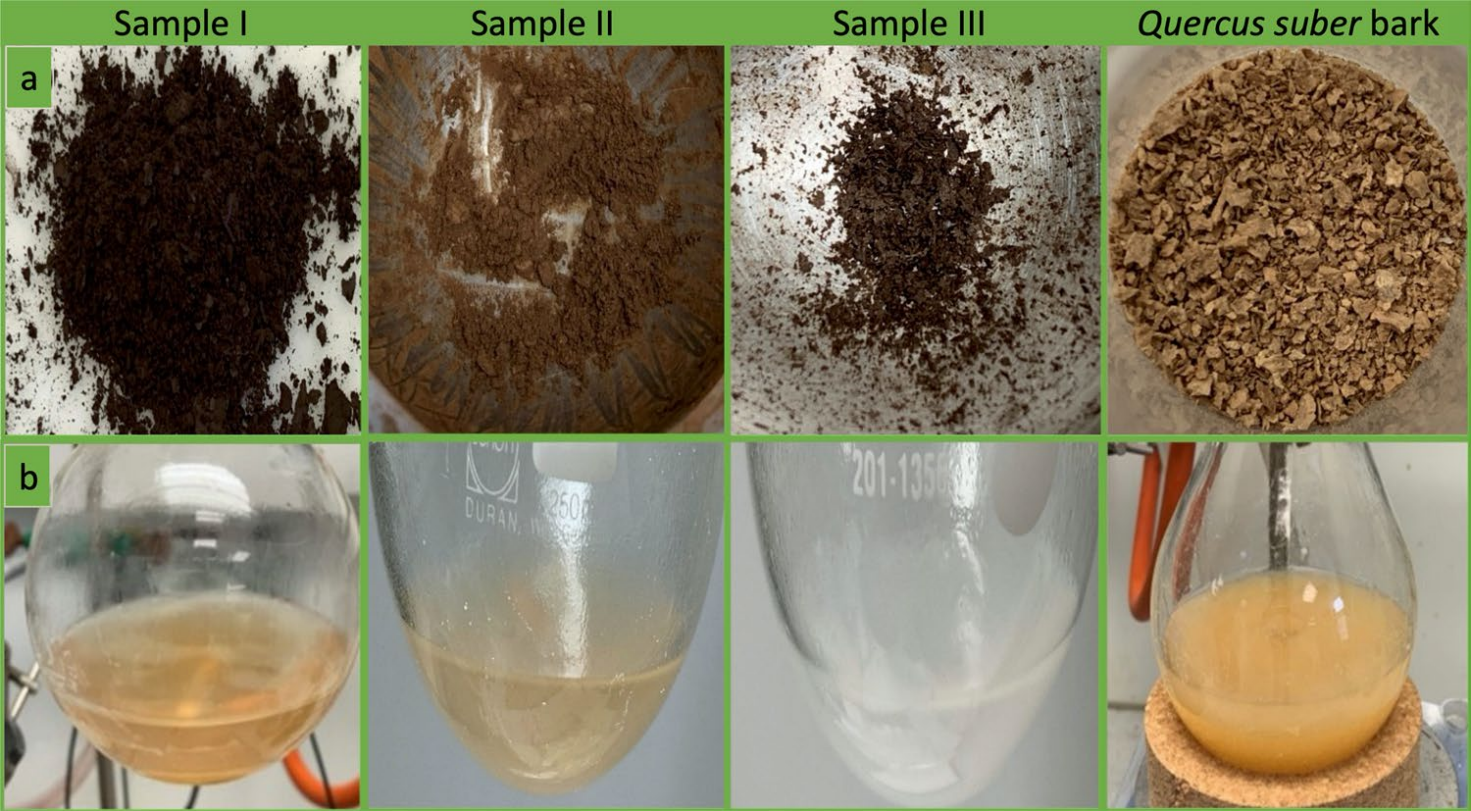
Appearance of the milled samples and extracts. (**a**) Samples after milling. (**b**) Dichloromethane extracts obtained after methanolysis and acidification to pH 6. Note the similarity in color between sample I (the putative fossil monkeyhair bark) and the sample of recent bark of *Quercus suber*, in comparison to samples II and III.

Three tissue samples from the two layers of the fossil monkeyhair tree (Fig. 1) as well as a recent control sample from the bark of *Quercus suber* were analyzed. The outer layer of the fossil (sample I) was interpreted as bark, the inner layer as degraded wood (sample II), and sample III as strands of laticifers. After milling the samples, they were found to be variously colored (Fig. 2). Subsequent methanolysis and acidification to pH 6 yielded dichloromethane extracts, that showed similar color for sample I (the putative fossil bark layer of the fossil monkeyhair tree) and the bark sample of *Quercus suber* (Fig. 2), while the extracts of samples II and III had different hues.

### Development and validation of HPLC–ESI–MS method

**Figure 3:**
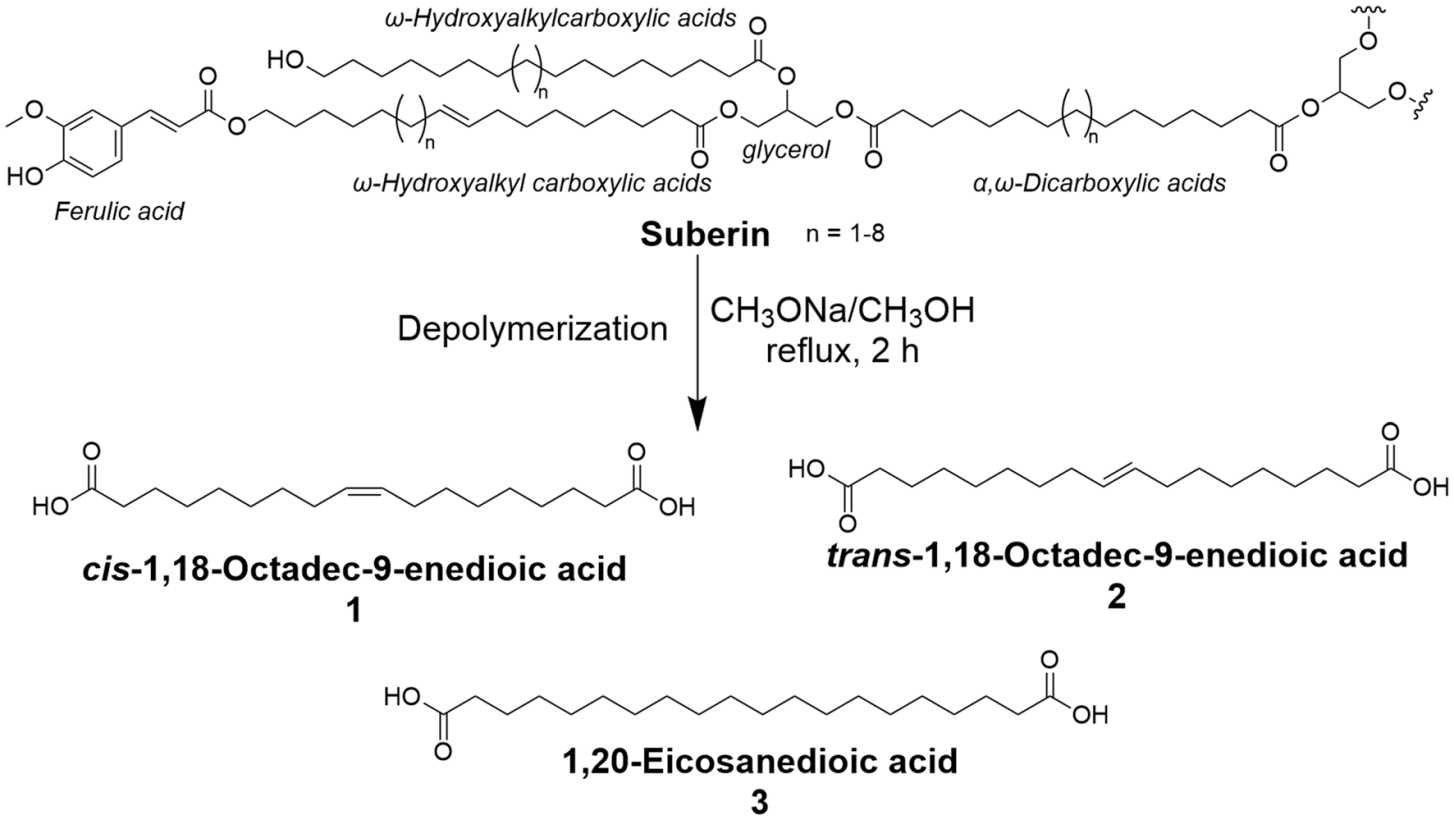
Suberin and selected constituents. Partial structure of the polymer suberin and structures of selected constituents **1**/**2** and **3** that are obtained after depolymerization of suberin using sodium methanolate in methanol.

An HPLC–ESI–MS method was developed and optimized for the analysis of selected constituents of suberin, the long-chain fatty acids 1,18-octadec-9-enedioic acid (mixture of *cis*- and *trans*-isomers, **1** and **2**) and 1,20-eicosanedioic acid (**3**) (Fig. 3). For the mobile phase we utilized methanol/water (1:1) containing 2 mmol/l of ammonium acetate, applying a linear gradient to 100% methanol/2 mmol/l ammonium acetate for the recovery of long-chain fatty acids **1**–**3** (for details see Materials and Methods). The method was validated by evaluating linearity, limit of detection (LOD), limit of quantification (LOQ) and selectivity according to the International Council for Harmonization (ICH) guidelines. Linearity was determined in a wide concentration range of 0.01–1 µmol/L of compounds **1**/**2** and 0.0125–1 µmol/L of compound **3**. The correlation coefficient was found to be > 0.99 in all cases, indicating linearity (Table 1). LOD and LOQ were determined, which correspond to the minimum concentration that can be confidently detected and quantified, respectively. With this method, compounds **1**–**3** could be detected in nanomolar concentrations (Table 1).

**Table 1:** Method validation parameters for HPLC–ESI–MS determination of standard compounds.

Parameters	*cis*/*trans*-1,18-Octadec-9-enedioic acid (1/2)	1,20-Eicosanedioic acid (3)
Concentration range	0.01–1 µmol/l	0.0125–1 µmol/l
Linear regression and correlation coefficient (*r*^2^)	*y* = 13216*x* + 220.7 (*r*^2^ = 0.9997)	*y* = 36927*x*–480.2 (*r*^2^ = 0.9913)
Limit of detection (LOD)^a^ (pg/µl)	75.8	53.3
Limit of quantification (LOQ)^b^ (pg/µl)	223	124

^a^The limit of detection was defined as the concentration that produced a signal-to-noise ratio of 3. ^a,b^For more details on the determination of limit of detection and quantification, see Materials and Methods.

### Optimization of methanolate-induced cleavage of suberin using recent bark

The method used for the determination of suberin constituents from *Quercus suber* bark^1^ was adapted to analyze the small amounts of sample material (ca. 100–250 mg) in this study. Sequential Soxhlet extraction with dichloromethane followed by methanol was used to remove waxes, non-polar constituents, and phenolic/polyphenolic compounds^1^. The samples were then treated with sodium methanolate in methanol to cleave the ester groups of suberin, followed by acidification to pH 6 and extraction with dichloromethane^1,19^. The extracts obtained were dried and weighed. After methanolate-induced cleavage and extraction, a *Quercus suber* bark sample (250 mg) yielded 41.9% (w/w) of the original mass and contained the analyzed hydrolysis products of suberin (Table 2). This result was comparable to previously reported yields of 40–50%^1^ which indicate that the extraction method could be applied to small amounts of sample. The dried extracts were transferred into a mixture of dichloromethane/methanol (3:1, v/v) for subsequent HPLC–ESI–MS analysis.

**Table 2:** Amounts of the suberin constituents *cis*-/*trans*-1,18-octadec-9-enedioic acid (**1**/**2**) and 1,20-eicosanedioic acid (**3**) detected in the samples.

Sample	Percentage (w/w) of extractives obtained after Soxhlet extraction, based on weight of untreated material	Percentage (w/w) of dried extract obtained after methanolate-induced cleavage, based on weight a) of untreated material b) after Soxhlet extraction	Amount of compound a) mg per g of untreated material b) mg per g of residue after methanolysis	
			Compounds 1 and 2	Compound 3
Cellulose thimble (Negative control)	1.6%	(a) 3.9% (b) 4.1%	< LOD	< LOD
*Quercus suber* bark (Positive control)	6%	(a) 33.5% (b) 41.9%	(a) 0.318 (b) 0.764	(a) 0.146 (b) 0.353
Sample I (Outer layer interpreted as fossil bark)	40%	(a) 15.7% (b) 26.3%	(a) 0.148 (b) 0.941	(a) 0.0777 (b) 0.493
Sample II (Inner tissue interpreted as degraded fossil wood)	73%	(a) 1.3% (b) 4.9%	< LOD	< LOD
Sample III (Inner tissue of fossil laticifers)	81%	(a) 12.8% (b) 66.7%	< LOD	< LOD

Amounts of compounds **1**/**2** and **3** are given as mg of compound per g of untreated material, and per g of residue obtained after methanolysis, respectively. The percentage (w/w) of residue obtained after methanolysis and extraction of fossil samples I–III, of the positive control (recent *Quercus suber*), and of the negative control (cellulose thimble used for extraction) were calculated and compared to the starting weight before sample preparation, and to the sample weight after Soxhlet extraction. The percentage (w/w) of extractives obtained after Soxhlet extraction in relation to the starting weight of the samples was additionally calculated.

### Methanolate-induced cleavage of fossil samples

The optimized extraction procedure and the validated HPLC–ESI–MS methods applied to the three tissue samples from the fossil monkeyhair tree yielded varying weights of material after Soxhlet extraction, followed by methanolate-induced cleavage. The weight of the samples obtained at each step are given in Supplementary Fig. S1.

### Qualitative analysis of suberin constituents

**Figure 4:**
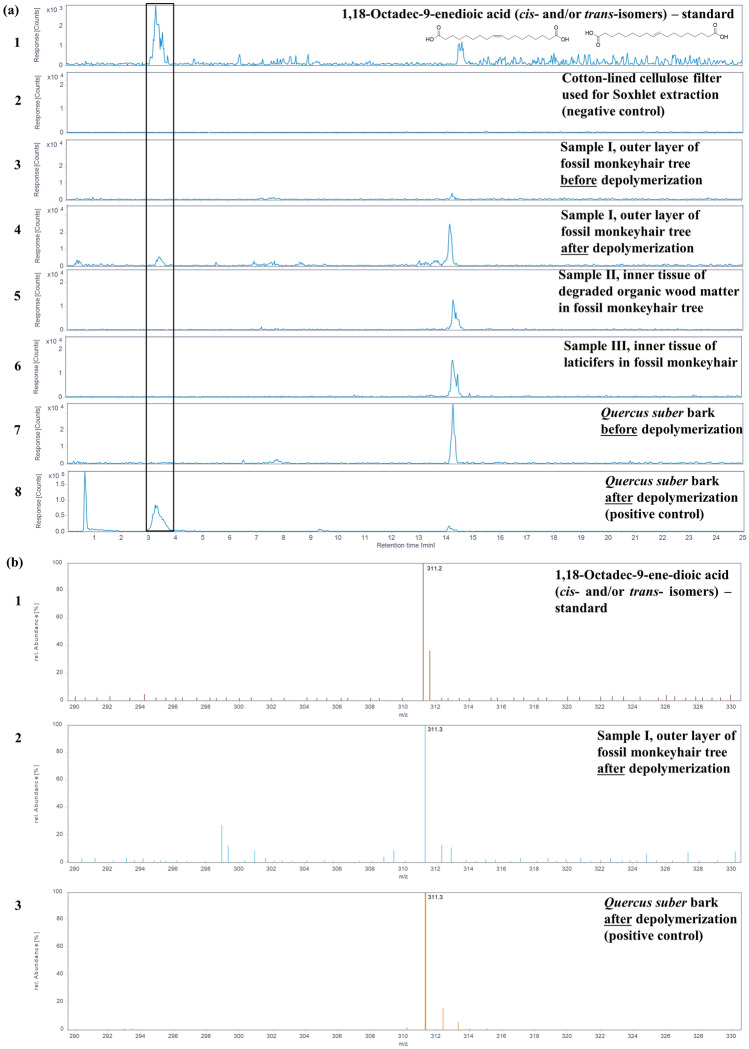
Presence of the suberin monomer 1,18-octadec-9-enedioic acid in sample I from the outer layer of the fossil monkeyhair tree after depolymerization and extraction detected by HPLC–ESI-MS. (**a**) Extracted-ion chromatograms of 1,18-octadec-9-enedioic acid (**1**/**2**, *m/z* 311.23 ± 0.70 Da for [M-H]^−^ in negative ion mode) of a standard solution of 1,18-octadec-9-enedioic acid (trace 1), from the extracts of the cotton-lined cellulose filter used for Soxhlet extraction (negative control, trace 2), sample I from the outer layer of fossil monkeyhair tree before depolymerization (trace 3), and after depolymerization (trace 4), sample II from the inner layer of the fossil monkeyhair tree (trace 5), and sample III from the inner layer of the fossil monkeyhair tree (trace 6), *Quercus suber* bark (positive reference for suberin, before depolymerization, trace 7), and *Quercus suber* bark (after depolymerization, trace 8). A signal for compounds **1/2** was detected with confidence in samples from *Quercus suber* and in sample I from the outer layer of the fossil monkeyhair tree. (**b**) Electrospray ionization mass spectra of standard 1,18-octadec-9-enedioic acid (**1**/**2**) at a retention time of 3.3 min (trace 1), extracts of *Quercus suber* bark after depolymerization at a retention time of 3.3 min (trace 2), and the peak in extracts of sample I from the outer layer of the fossil monkeyhair tree after depolymerization at a retention time of 3.4 min (trace 3), showing the deprotonated molecule [M-H]^-^ at *m/z* 311.2 ± 0.3 detected in the negative ion mode.

**Figure 5:**
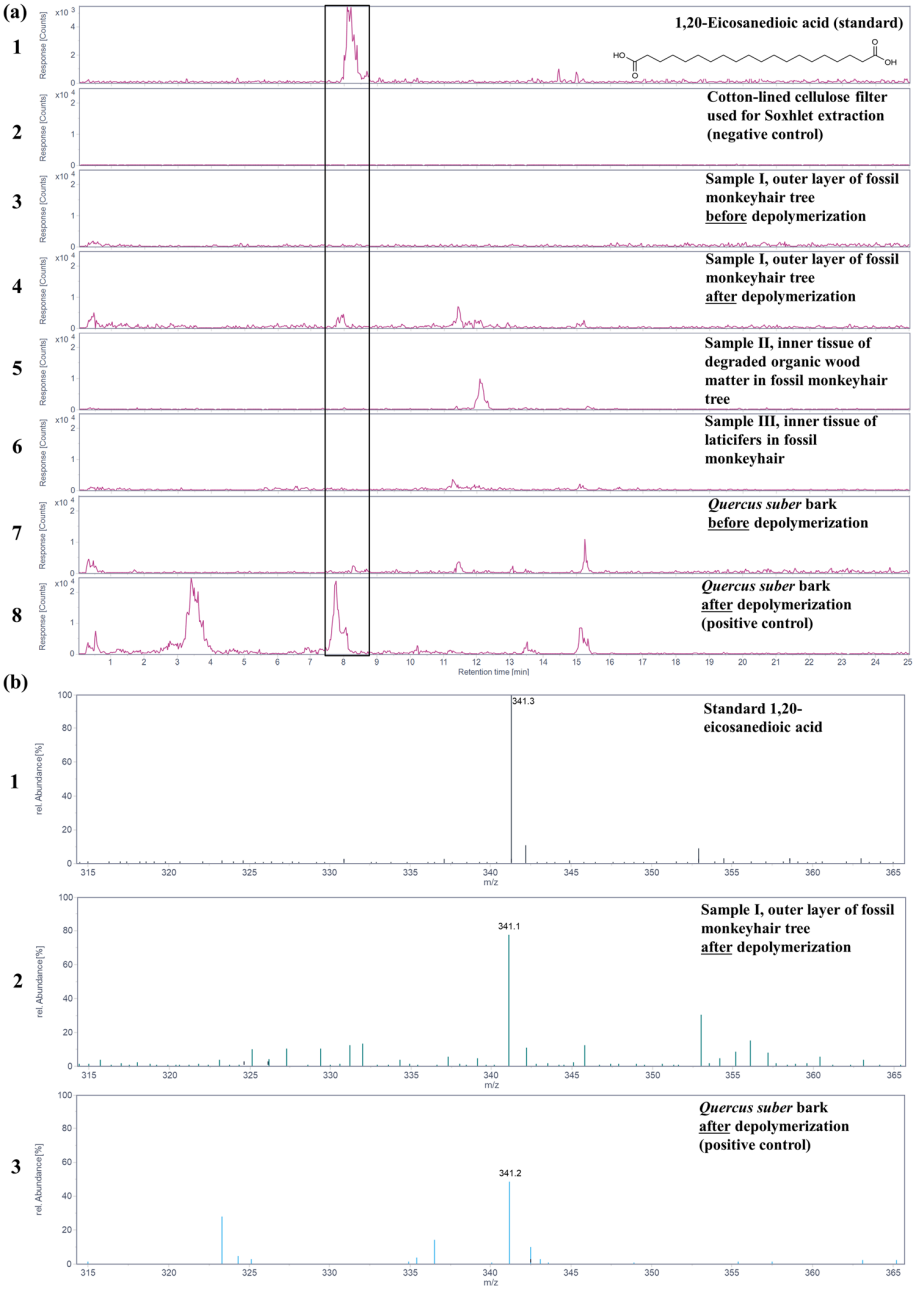
Presence of the suberin monomer 1,20-eicosanedioic acid in sample I from the outer layer of the fossil monkeyhair tree after depolymerization and extraction detected by HPLC–ESI–MS. (**a**) Extracted-ion chromatograms of 1,20-eicosanedioic acid (**3**, *m/z* 341.23 ± 0.70 Da for [M-H]^−^ in negative ion mode) of a standard solution of 1,20-eicosanedioic acid (trace 1), from the extracts of the cotton-lined cellulose filter used for Soxhlet extraction (negative control, trace 2), sample I of putative bark from the outer layer of the fossil monkeyhair tree before depolymerization (trace 3) and after depolymerization (trace 4), sample II of putative wood from the inner layer of the fossil monkeyhair tree (trace 5), sample III of laticifers from the inner layer of the fossil monkeyhair tree (trace 6), *Quercus suber* bark (positive control for suberin) before depolymerization (trace 7) and after polymerization (trace 8). Compound **3** was detected with confidence in samples from the *Quercus suber* bark and in sample I of putative bark from the outer layer of the fossil monkeyhair tree. (**b**) Electrospray ionization mass spectra of standard 1,20-eicosanedioic acid (**3**) at a retention time of 8.0 min (trace 1), in sample I from the outer layer of the fossil monkeyhair tree.after depolymerization at a retention time of 7.9 min (trace 2) and of extracts from *Quercus suber* bark after depolymerization at a retention time of 7.8 min (trace 3), showing the deprotonated molecule [M-H]^−^ at *m/z* 341.2 ± 0.3 detected in the negative ion mode.

The characteristic constituents of suberin, after methanolate-induced cleavage, were found in the extracts from the outer layer of the fossil monkeyhair tree. To confirm the identity of the compounds, the extracted ion chromatograms (EIC) for the deprotonated molecular ions (Fig. 4a, trace 4 and 5a, trace 4), and electrospray ionization mass spectra (ESI-MS) in negative mode at the respective retention times of the constituents (Fig. 4b, trace 2 and 5b, trace 2) were compared to the positive controls. They matched with solutions of 1,18-octadec-9-enedioic acid (Fig. 4a, trace 1; Fig. 4b, trace 1) and 1,20-eicosanedioic acid (Fig. 5a, trace 1 and 5b, trace 1) used as standards for comparison, as well as to those obtained from *Quercus suber* extracts (Fig. 4a, trace 8 and 5a, trace 8)*.* In the negative control, utilizing the same extraction procedure with a Soxhlet sleeve consisting of cellulose, none of these compounds were detected (Fig. 4a, trace 2 and 5a, trace 2). These results further confirm that the peaks observed in sample I, i.e., from the outer layer, are suberin, which indicates that the outer layer is bark, and that these compounds were neither detected in the tissues of sample II (Fig. 4a, trace 5 and 5a, trace 5) nor in sample III (Fig. 4a, trace 6 and 5a, trace 6) of the inner layers that are interpreted as degraded fossil wood and laticifers, respectively.

Moreover, prior to depolymerization induced by methanolate, the suberin constituents 1,18-octadec-9-enedioic acid (Fig. 4a, trace 3) and 1,20-eicosanedioic acid (Fig. 5a, trace 3) were not detected in methanolic extracts, indicating that these carboxylic acids had been present as constituents of the polymeric suberin structure and were released upon depolymerization. This provides indirect evidence for corroborating the presence of suberin in sample I of putative bark from the outer layer of the fossil monkeyhair tree.

### Quantitative analysis of suberin constituents

HPLC–(DAD-UV)-ESI-MS analyses were undertaken on the weighed dried extracts of the samples after methanolate-induced cleavage, and of the weighed dried extracts obtained after Soxhlet extraction (containing the extractives). For quantitative analysis, the peak areas from the extracted ion chromatograms were calculated, and the concentrations of the selected compounds in the extracts were determined utilizing the calibration curves that were previously measured (see Table 1). The following parameters were calculated: (1) Percentage (w/w) of extractives obtained after Soxhlet extraction (based on weight of untreated material) and (2) percentage (w/w) of dried extract obtained after methanolate-induced cleavage (based on weight of untreated material and on weight after Soxhlet extraction). These were calculated according to Eqs. (1) and (2) and are given in Table 2 for each sample.

1 $$Percentage \; \left(w/w\right) \; of \; extractives= \frac{Weight \; after \; Soxhlet \; extraction}{Weight \; of \; untreated \; material}\times 100$$

2 $$Percentage \; \left(w/w\right) \; of \; residue= \frac{Weight \; after \; methanolate-induced \; cleavage}{Weight \;of \; untreated \; material \; or \; after \; Soxhlet \; extraction}\times 100$$

To normalize the data for comparison, the amounts of the detected compounds were calculated in mg per g of residue obtained after methanolate-induced cleavage, and per g of starting material, respectively (Table 2).

Sample I (109 mg) from the outermost layer, the tissue interpreted to be fossil bark, lost 40% (w/w) of its initial sample weight during Soxhlet extraction (Table 2 and Supplementary Fig. S1). This is a significantly higher percentage than that observed for the *Quercus suber* bark (6%, Table 2 and Supplementary Fig. S1) and may be due to chemical changes that occurred during fossilization, which may have altered its chemical composition and led to the accumulation of smaller, non-polymerized compounds. After methanolate-induced cleavage of the extractive-free material, 26.3% (w/w) relative to the weight of the original material were obtained, which is only 1.6-fold less than the amount obtained from the recent *Quercus suber* control sample in this step (Table 2).

Extracts of sample II (187 mg) from the inner layer interpreted as the degraded remains of fossil wood showed an even higher percentage of extractives (74%, Table 2). After methanolate-induced cleavage of the sample (51 mg), only a low percentage of residue was obtained (4.9%, Table 2 and Supplementary Fig. S1). This suggests that this layer does not contain many hydrolyzable polymers.

The laticifers from the inner layer (sample III, 12.5 mg) showed the highest percentage of extractives in the fossil, constituting 81% of the sample (Table 2). After methanolate-induced cleavage of the extractive-free material (2.4 mg), a very low amount of residue was obtained (1.6 mg), accounting to 66.7% (w/w) compared to the weight after Soxhlet extraction, or to 12.8% (w/w) of the weight of dry, untreated fossil material (Table 2 and Supplementary Fig. S1). The high proportion of extractives present in the fossil is likely due to the presence of various hydrocarbon derivatives, e.g., terpenoid compounds, phenolic compounds, and polycyclic hydrocarbons, as a result of diagenesis^6,9,11,12,14,20^.

Regarding fatty acid composition, only in the extracts of *Q. suber* bark and in sample I, interpreted as bark of the fossil monkeyhair tree, the amounts of 1,18-octadec-9-enedioic acid (**1**/**2**) and 1,20-eicosanedioic acid **(3)** were above the LOQ (Table 2) with a signal-to-noise ratio of over 10, permitting quantification with high confidence according to the International Council for Harmonization (ICH) guidelines. This was not the case for the extracts of the inner tissues of the fossil monkeyhair tree, which had been interpreted as degraded wood (sample II) and laticifers (sample III), in which the analytes were below the limits of detection and quantification with a signal-to-noise ratio below 3 (Table 2).

The amount of 1,18-octadec-9-enedioic acid in sample I of putative bark from the outermost layer of the fossil monkeyhair tree per g of untreated material was approximately 50% of the amount found in *Quercus suber* bark. Similarly, the amount of 1,20-eicosanedioic acid was approximately 50% of the amount found in *Quercus suber* bark per g of untreated material (Table 2).

### Fluorescence spectroscopy

**Figure 6:**
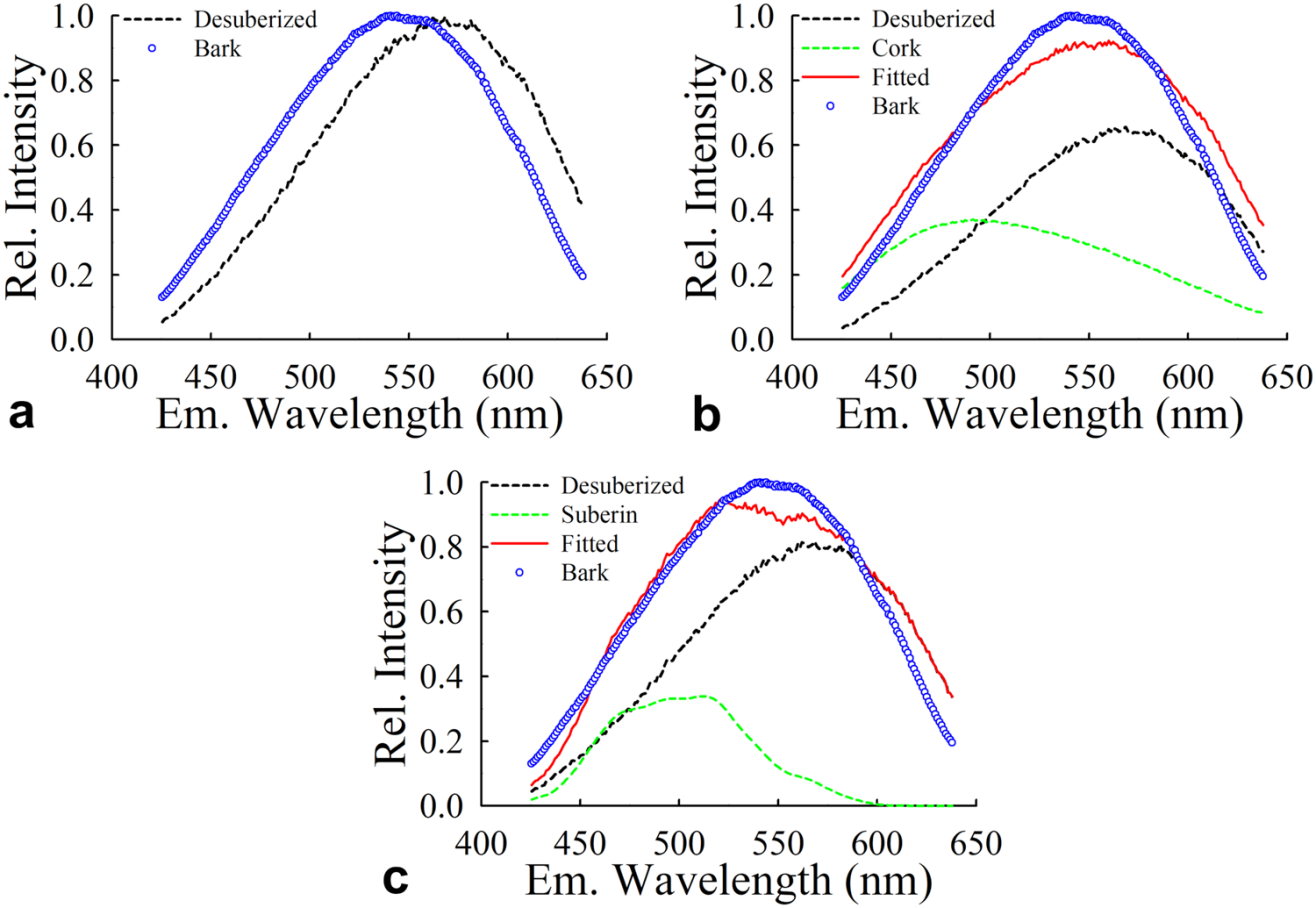
Curve fitting-based assessment of the species contributing to the average fluorescence emission spectrum of sample I from the outermost layer of the fossil monkeyhair tree obtained upon two-photon excitation with ultrashort pulses of light with 790 nm wavelength. The figure illustrates the procedure for determining the constituents of sample I through curve fitting of its average emission spectrum using different models. (**a**) The average emission spectrum of untreated sample I (labeled “Bark” in the legend) is plotted alongside the average emission spectrum obtained from multiple scans of desuberized sample I (labeled “Desuberized bark” in the legend). To quantify the disparity between untreated sample I and desuberized sample I, the residual sum of the squared differences (*RSS*) between the two spectra was computed, resulting in a value of *RSS* = 5.4. (**b**) The average emission spectrum of untreated sample I was fitted, by minimizing RSS, with a model consisting of a weighted sum of two individual emission spectra, as defined by the equation ${I\left(\lambda \right)}_{Bark}=a\cdot {I\left(\lambda \right)}_{Desuberized}+b \cdot {I\left(\lambda \right)}_{Cork}$, where ${I\left(\lambda \right)}_{Cork}$ is the average emission spectrum obtained from a sample of recent *Quercus suber* bark. During this fitting process, the weights (*a, b*) assigned to each component are adjusted to minimize the mismatch between untreated sample I and the fitted curve, resulting in an *RSS* value of 1.3. (**c**) The same fitting approach as in (**b**) was applied using an average spectrum from desuberized sample I and a previously reported emission spectrum of suberin, acquired using a 790 nm excitation wavelength (labeled “Suberin” in the legend) instead of the cork spectrum. This fitting yields an *RSS* value of 1.0.

Two-photon microspectroscopy scans^16,17^ were recorded using an excitation wavelength of 790 nm on three distinct samples: untreated material of sample I from the outermost layer of the fossil monkeyhair tree, a “desuberized” portion of sample I (residue after suberin hydrolysis and extraction of constituents), and *Quercus suber* bark as a control sample. The emission spectrum for each sample was obtained by averaging the emission spectra of all pixels contained within a region-of-interest (ROI), then averaging the ROI-level emission spectra from multiple scans obtained for that specific sample. Figure 6 presents the average spectrum of untreated sample I, the outermost layer of the fossil monkeyhair tree, and its fitting with three different “theoretical” models: (a) a model consisting of the spectrum of the desuberized sample alone; (b) a model consisting of a weighted sum between the desuberized outer layer’s spectrum with the average spectrum measured from the control sample of *Quercus suber* bark containing 40–50% suberin as well as other materials^18^, some of which, such as lignin, also fluoresce^21^, and (c) a model consisting of the weighted sum of the desuberized sample spectrum with the previously reported suberin emission spectrum obtained from the root endodermis of *Arabidopsis thaliana*^22^. The data fitting strategy consisted of adjusting the weights (or relative amplitudes) of the different spectral components to minimize the residual sum of squared differences (*RSS*). The fitted curves yielded *RSS* values of 1.3 or and 1.0 when the cork (suberin plus other materials) or the suberin spectra were included, respectively, compared to an *RSS* value of 5.4 when the suberin component was excluded from the fit.

The model that incorporated the emission spectra of both the desuberized sample and the suberin, obtained from either the control sample of recent *Quercus suber* bark (Fig. 6b) or the previously reported emission spectrum of suberin, resulted in significantly better fit spectrum of suberin (Fig. 6c). This can be attributed to the appreciable amount of suberin found in *Quercus suber*, which suggests that the observed differences between the emission spectra of the untreated sample I and desuberized sample I were likely due to the presence of suberin in the untreated sample I, the outermost layer of the fossil monkeyhair tree interpreted as bark. Therefore, incorporating the emission spectrum of a suberin-containing sample into the model was shown as an important factor that improved the fitting. Despite this, the best fit spectrum obtained here was only an approximation and not an exact match to the emission spectrum of the untreated sample I. This suggests the presence of other contributing factors, such as the inherent variation in suberin emission spectra across different species, and the potential impact of other plant materials such as lignin on fluorescence due to their structural or intrinsic properties. Notably, the emission spectrum of lignin, when excited at 790 nm, significantly overlaps with that of suberin. However, although the model utilizing cork rather than pure suberin is still a very good fit, it is slightly less so than the model utilizing suberin and the desuberized fossil material. This further suggests that the addition of lignin to the model weakens rather than strengthens it supporting the interpretation that it is suberin rather than another plant material such as lignin that provides the suberin-like component of the fluorescence emission spectrum of untreated sample I. Complex interactions among various components could collectively contribute to moderate, but observable differences between fitted and measured outermost layer spectra.

## Discussion

Among the three tissues sampled from the fossil monkeyhair tree, the polymeric constituent suberin occurs only in one tissue of a single layer − the outer layer that has been morphologically interpreted as bark. Suberin was not found in the tissues of the inner layer thought to pertain to wood and laticifers. Specifically, the distinctive suberin constituents 1,18-octadec-9-enedioic acid and 1,20-eicosanedioic acid were detected after alkaline hydrolysis of the polymer. The preservation of suberin in its intact polymeric form was indirectly confirmed by analyzing the samples before and after depolymerization. Furthermore, two-photon fluorescence microspectroscopy^16,17^ supported the occurrence of intact suberin in the tissue of the outermost layer through its characteristic autofluorescence^22^.

Interestingly, the tissue from the outermost layer of the fossil monkeyhair tree (sample I) contained all of the suberin constituents targeted. In hydrolysates of the inner layer, both the degraded material of putative wood (sample II) and the naturally vulcanized laticifers (sample III), no suberin constituents were detected. Our study confirms that only the outermost layer (sample I) could be chemically characterized as bark. Moreover, detection of the characteristic long-chain di-carboxylic acids only after the depolymerization process implies that intact, polymeric suberin was preserved in the monkeyhair tree bark. This is further supported by the results of the fluorescence spectroscopy analysis.

The percentage of residue of the suberin constituents in sample I of bark from the outermost layer of the fossil monkeyhair tree (26.3% w/w) was quite high and comparable to that of the bark from the cork oak *Q. suber* (40–50% w/w). Since monkeyhair trees are extinct and there are no data available on the suberin content of the bark in this kind of tree, we cannot assess how much of the suberin originally present in the tree bark was preserved over the course of 45 million years. Nevertheless, it is possible that the suberin content in the bark of the then-living monkeyhair tree was different from that found in the bark of living *Quercus suber*, since the amount can be expected to be species-specific. It is well-known that the thick bark of *Quercus suber* has particularly high amounts of suberin (40–50% w/w) when compared to that of other taxa trees^23^. Partial degradation of suberin and other cell-wall polymers, e.g., hemicellulose^10^, present in the once-living monkeyhair tree due to diagenesis is not excluded, as indicated by the high percentage of extractives we found in the outer layer of the fossil monkeyhair tree, and by the fact that the amounts of 1,18-octadec-9-enedioic acid and 1,20-eicosanedioic acid in the outermost layer of the fossil were approximately 50% of their amounts in the control sample of *Quercus suber* (Table 2). Fatty acid constituents of suberin released after degradation might undergo decarboxylation to aliphatic alkanes or alkenes, which may explain why we did not detect the fatty acids **1**–**3** before methanolate-induced cleavage of the fossil material^24–27^. After hydrolysis of suberin to release its fatty acid constituents, decarboxylation may have occurred under specific conditions of fossilization, converting the constituents to straight-chain alkanes and alkenes, as observed for the fossil *Diaphorodendron* sp. bark (estimated to be 298–358 million years old) following analysis by pyrolysis gas chromatography (GC)/MS^16,17^.

Raman spectroscopy studies of the latex-bearing laticifers (i.e., monkeyhair) and the embedding lignites at Geiseltal preserving the Eocene monkeyhair tree axes show that the plant material had not been subjected to temperatures above 100 °C^15^; the triterpenoids described in a previous study^14^, as well as the fatty acids identified in the present study could not have survived temperatures above 50–60 °C.

Up to now, the interpretation of the outer layer of the woody axes of the Eocene monkeyhair tree as bark^6–8^ has been primarily based on morphological analysis and micro-CT. Here, our study on the analysis of different tissues of a monkeyhair tree using HPLC–ESI-MS provides the first chemical evidence for the presence of suberin supporting previous interpretations of the outer tissue as bark. Our data also show that the two tissues of the inner layer do not contain suberin and may indeed represent the degraded organic remains of wood and laticifers, as had been proposed^6^. Based on nuclear magnetic resonance spectroscopy (NMR), GC/MS, pyrolysis GC/MS, and Raman spectroscopy, the fossilization of the monkeyhair laticifers through natural, low-temperature vulcanization of the latex has become clear^6,11–15^.

The few previously published studies on the chemical characterization of fossil conifer bark have not detected suberin^24,25,28,29^. Despite the variety of applied methods, which included scanning electron microscopy, Fourier-transform infrared spectroscopy (FTIR), UV spectroscopy, GC, and GC/MS, neither suberin nor its fatty di-acid constituents were detected in their fossil bark specimens, not even in trace amounts^25^. Only the presence of holocellulose, carboxylic acids, and lignin were reported^24,25,28,29^. The high content of free carboxylic acids found in their specimens before alkaline hydrolysis, in comparison to the control, may mean that suberin had already been degraded in their samples. There was little information on the taxonomic determination of the wood, morphological analysis of the bark, or geological age of the fossil deposits available in these studies. In contrast, the fossil wood in the Eocene Geiseltal deposit, based upon identification of distinctive resinites, is from four taxa: the monkeyhair tree *Coumoxylon hartigii*^7^, conifer family Cupressaceae (*Taxodium* sp.), angiosperm family Burseraceae, and angiosperm Dipterocarpaceae family^20^.

Conversely, the tree axes of the monkeyhair tree of the Eocene deposit in Geiseltal have been subject to intense geological, paleontological, and chemical study for the last 175 years. In the last four decades, the bark and inner tissues in the woody axes have been extensively investigated through morphological analysis, NMR, GC/MS, pyrolysis GC/MS, micro-computer tomography (CT), and Raman spectroscopy^6,11,15^. Most of these analyses have concentrated on confirming and characterizing the latex of the inner layer. Our chemical analysis, using HPLC–ESI-MS, specifically examined the outer tissue supporting interpretation of this tissue as bark based on the detection of suberin constituents. Hence, our study presents the first chemical evidence for intact suberin in fossil bark, from a 45-million-year-old monkeyhair tree from the middle Eocene Geiseltal deposit in Germany. Fluorescence emission spectra also supported the presence of suberin.

## Conclusions

Unambiguous chemical evidence for the presence of intact suberin, a hallmark constituent of bark, in the outer layer of a tree axis of the fossil monkeyhair tree from the middle Eocene Geiseltal deposit in eastern Germany is presented. Analytical HPLC–ESI-MS studies confirmed that the outer layer of a fossil monkeyhair tree sample was bark, while the two tissues comprising the inner layer did not contain suberin. This is the first study to provide chemical evidence of suberin in the bark of fossil tree trunks, which was also supported by fluorescence spectroscopy. Based on our present results and previous data from Raman spectroscopy, fossilization conditions in the Eocene Geiseltal deposit appear to have been mild, with low moisture and low temperatures, contributing to the remarkable preservation of bark and inner laticifer mats of the monkeyhair trees that grew there 45 million years ago.

## Materials and methods

### Fossil tissue samples

The fossil monkeyhair tree sampled was collected from the brown coal mines of the middle Eocene Geiseltal deposit near Halle (Saale), eastern Germany, sometime during active mining between 1920 and 2000. This specimen is deposited in the Geiseltal Collections of the Martin Luther University Halle-Wittenberg under inventory number GMH Y74. Samples were taken of three tissues for analysis. The first tissue, sample I, came from the outer layer, the putative bark^6^. The other two tissues were taken from the inner layer; sample II is the degraded organic matter believed to represent the remnants of wood, while sample III is composed of naturally vulcanized latex of the laticifers^6^. The central space in the woody axis consisting of a hollow area with small fragments of mudstone^6^ did not contain any original tissues from the plant and thus was not sampled for this study.

### Control materials

Recent bark from a living tree of *Quercus suber*, the cork oak, was obtained for method development and as a positive control. The piece of outer bark was collected in the Peneda-Gerês National Park in northern Portugal in September 2019. As a negative control, a cellulose sleeve for Soxhlet extraction (Cytiva Europe GmbH/GE Healthcare, Freiburg i. Br., Germany) without any contact to the samples was used.

### Chemical reagents

Typical, abundant suberin constituents were selected for use as standards: the fatty acids 1,18-octadec-9-enedioic acid (a mixture of *cis*- and *trans*-isomers, **1**/**2**) and 1,20-eicosanedioic acid (**3**). The compounds were purchased from abcr GmbH, Karlsruhe, Germany. All HPLC solvents and mobile phase additives (formic acid, acetic acid, and ammonium acetate) utilized for analysis were of LC–MS grade. Water was obtained from an in-house Millipore milliQ water purification system.

### Sample preparation

The bark of *Quercus suber* (1.58 g) was cut into pieces, frozen in liquid nitrogen, then ground using a Retsch MM40 mixer mill (Retsch GmbH, Haan, Germany). The material was subjected to Soxhlet extraction (size 29/32, Lenz Laborglas GmbH, Wertheim, Germany) using 150 ml of dichloromethane for 6 h followed by 150 ml of methanol for 6 h. Then, the remaining solid material was left to dry under a hood for 24 h. Samples I (183 mg), II (187 mg), III (12.5 mg), and the negative control (1.93 g) were treated using the same procedure.

### Methanolate-induced cleavage and extraction of suberin constituents

The method developed by Pereira^1^ for the cleavage and extraction of suberin constituents from *Quercus suber* bark was adapted for small amounts of sample material. Dry material (0.25 g) obtained after extraction was refluxed with 3% sodium methoxide in dry methanol (25 ml) for 2 h with stirring under an argon atmosphere. The resulting mixture was filtered, and the filtrate was subsequently acidified to pH 6 using 2 mol/l sulfuric acid in methanol (ca. 2 ml, accurately determined by a pH meter). The filtrate was evaporated to dryness using a rotary evaporator at about 40 °C under reduced pressure. The residue was suspended in 50 ml of distilled water and extracted three times with 50 ml of dichloromethane each. The combined organic extracts were dried over anhydrous magnesium sulfate (ca. 5 g), filtered, and evaporated to dryness. The residue was weighed, and aliquots were taken for LC–MS analysis. Methanolysis and extraction were similarly performed using samples I (109 mg), II (51.0 mg), III (2.40 mg) and the cellulose sleeve used for Soxhlet extraction (negative control, 1.9 g). The solid residue after methanolysis and extraction of suberin constituents was used as “desuberized” sample for fluorescence spectroscopy.

### HPLC–(DAD-UV)-ESI-MS analysis

Aliquots from the residues obtained after methanolysis were dissolved in a 3:1 (v/v) mixture of methanol/dichloromethane and subsequently analyzed by HPLC–(DAD-UV)-ESI-MS. Measurements were performed on an Agilent 1260 Infinity HPLC instrument coupled to an Agilent Infinity Lab LC/MSD Single Quadrupole mass spectrometer with an electrospray ion source and a DAD-UV detector (200–600 nm). Chromatographic separation was performed on an EC 50/3 Nucleodur C18 Gravity, 3 μm (Macherey–Nagel, Dueren, Germany). Mobile phase A consisted of methanol with 2 mmol/l ammonium acetate, and mobile phase B consisted of water with 2 mmol/l ammonium acetate. The run started with 50% A and 50% B for 1 min, followed by a gradient that reached 100% of eluent A after 15 min. Subsequently, the column was flushed for 10 min with 100% of mobile phase A, then with 50% A and 50% B for 5 min before starting the next run. Positive and negative full scan MS was obtained from 100 to 1000 m/z. The column temperature was set at 40 °C, the injection volume was 5 μl, and the flow rate was adjusted to 0.5 ml/min. Using this method, the retention time of 1,18-octadec-9-enedioic acid was 3.33 min (*cis*/*trans*-isomers were not separated), and that of 1,20-eicosanedioic acid (**3**) was 7.89 min.

### Qualitative and quantitative analysis

Identification of the peaks was performed using the Data Analysis program on OpenLab CDS 2.4 software (Agilent Technologies Germany GmbH & Co. KG, Waldbronn, Germany). The EIC was used to evaluate peak areas and provide a quantitative estimate of the detected compounds according to the following parameters to target the deprotonated molecules: 1,18-octadec-9-enedioic acid (*m/z* 311.23 ± 0.70) and 1,20-eicosanedioic acid (*m/z* 341.23 ± 0.70).

Limit of detection (LOD) and limit of quantitation (LOQ) were determined experimentally by residual standard deviation of regression, and resulting signal-to-noise ratios from the calibration curve (6-sigma method) in accordance with the International Council for Harmonization (ICH) guidelines. Resulting signals from samples with known low concentrations of analyte were compared with those of blank samples, establishing the minimum concentration at which the analyte could be reliably detected. The signal-to-noise ratio of the instrument was calculated using OpenLab CDS software 2.4 applying the 6-sigma algorithm, which calculates the ratio of peak height to noise of the closest range. A signal-to-noise ratio of 3 was considered acceptable for estimating the LOD, and a signal-to-noise ratio of 10 was acceptable for estimating the LOQ. LOD and LOQ of the compounds were calculated to be: 1,20-eicosanedioic acid, LOD: 53.3 pg/µl; LOQ: 124 pg/µl; 1,18-octadec-9-enedioic acid, LOD: 75.8 pg/µl; LOQ: 223 pg/µl.

### Fluorescence spectroscopic analysis

Fluorescence images of three of the same samples used in the chemical analyses above were taken using two-photon microspectroscopy. They were acquired using a spectrally resolved two-photon optical microspectroscope^16^ consisting of a tunable femtosecond laser (MaiTai™, Spectra Physics, Santa Clara, CA), an inverted microscope (Nikon Eclipse Ti™, Nikon Instruments Inc., Melville, New York, USA) equipped with an infinity-corrected, plan apo objective lens (60 × , NA = 1.4, WD = 0.13 mm; Nikon Instruments Inc.), an OptiMiS scanning/detection head (Aurora Spectral Technologies, Grafton, WI) and an electron multiplying CCD (EMCCD) camera (iXon X3 897, Andor Technologies, Belfast, UK). The samples were scanned using a line-shaped excitation beam with a power of 0.35 mW/voxel and an integration time of 35 ms per pixel. The line-shaped beam was swept over a field-of-view corresponding to an area in the sample of 117 µm × 80 µm. Each individual scan produced a set of 200 two-dimensional fluorescence intensity images (or wavelength channels), where each channel represents the pixel-level emission intensities for a different wavelength band, spanning the range between ~ 425 and ~ 645 nm, with ~ 1.1 nm bandwidth for each channel. In each scan, distinct regions of interest (ROIs) were identified and delineated, and the emission spectrum for a specific ROI was generated by averaging the emission spectra of all pixels contained within that ROI. Finally, the ROI-level emission spectra from multiple scans obtained from a particular specimen were averaged to determine a characteristic spectrum for the corresponding specimen. Scans were conducted at varying excitation wavelengths − from 750 to 790 nm, with 40 nm increments − for each field of view.

In addition, two-photon fluorescence microspectroscopy^17,18^ was used to investigate the characteristic autofluorescence of intact suberin in the same set of samples. The average fluorescence emission spectra were measured after two-photon excitation with ultrashort pulses of light with 790 nm wavelength, specifically on the outer layer of the fossil monkeyhair tree, desuberized material from this same layer (the solid residue after the extraction of suberin constituents as described above), and the bark of *Quercus suber* which consists largely of suberin but also contains other components such as lignin^18^. In addition, following reports of suberin autofluorescence under the same excitation conditions, modeling of the average fluorescence emission of the fossil material as a mixture of desuberized fossil material, pure suberin (values from literature), and suberin mixed with other natural bark material (*Quercus suber*) was carried out to investigate whether or not suberin autofluorescence is a component of the observed fossil fluorescence.

### Supplementary Information

Supplementary Figure S1.

## Data Availability

The authors declare that data supporting the findings of this study are available within this paper.

## Abbreviations

DAD-UV: Diode array detector with ultraviolet detection
EIC: Extracted ion chromatogram
FTIR: Fourier-transform infrared spectroscopy
GC/MS: Gas chromatography coupled to mass spectrometry
HPLC–ESI-MS: High performance liquid chromatography coupled to electrospray ionization mass spectrometry
ICH: International Council for Harmonization
LC–MS: Liquid chromatography coupled to mass spectrometry
LOD: Limit of detection
LOQ: Limit of quantitation
Micro-CT: High-resolution X-ray microcomputed tomography
*m/z*: Mass-to-charge ratio
NMR: Nuclear magnetic resonance spectroscopy
Pyrolysis GC/MS: Pyrolysis coupled to gas chromatography–mass spectrometry analysis
