# Supplementary material for: Suberin, the hallmark constituent of bark, identified in a 45-million-year-old monkeyhair tree (Coumoxylon hartigii) from Geiseltal, Germany
Source: Sci Rep. 2024 Jan 2;14:118. doi: 10.1038/s41598-023-50402-y (PMC10761729; doi:10.1038/s41598-023-50402-y)
Supplement: Supplementary file 1 — Supplementary Figure S1. [file 41598_2023_50402_MOESM1_ESM.pdf]

Supplementary Material

Suberin, the hallmark constituent of bark, identified in a 45-million-year-old monkeyhair tree (*Coumoxylon hartigii*) from Geiseltal, Germany

Mariam Tahoun[1], Carole T. Gee[2,*], Victoria E. McCoy[3], Michael Stoneman[4], Valerica Raicu[4,5], Marianne Engeser[6] & Christa E. Müller[1,*]

[1] PharmaCenter Bonn, Pharmaceutical Institute, Department of Pharmaceutical & Medicinal Chemistry, University of Bonn, An der Immenburg 4, D-53121 Bonn, Germany

[2] Institute of Geosciences, Division of Paleontology, University of Bonn, Nussallee 8, D-53115 Bonn, Germany

[3] Department of Geosciences, University of Wisconsin-Milwaukee, Milwaukee, Wisconsin 53211, USA

[4] Department of Physics, University of Wisconsin-Milwaukee, Milwaukee, Wisconsin 53211, USA

[5] Department of Biological Sciences, University of Wisconsin-Milwaukee, Milwaukee, Wisconsin 53211, USA

[6] Kekulé Institute for Organic Chemistry and Biochemistry, University of Bonn, D-53121 Bonn, Germany

* Corresponding authors:

Christa E. Müller: email, christa.mueller@uni-bonn.de; Carole T. Gee: email, cgee@uni-bonn.de

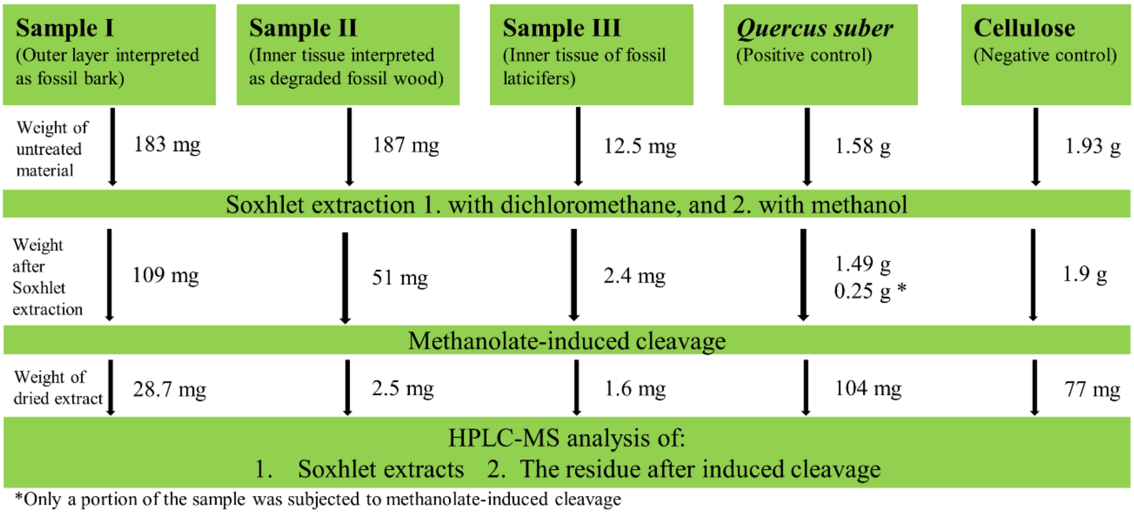

Supplementary Fig. S1. Workflow of Soxhlet extraction and methanolate-induced cleavage of the three tissue samples from the fossil monkeyhair tree, and the bark of *Quercus suber* (positive control), and the cellulose thimble (negative control). The weights of the untreated samples and of the samples obtained after each step are given.
